# Clinical characteristics of primary atrial tumor and their diagnostic value: A retrospective study of 10 years

**DOI:** 10.3389/fsurg.2023.1097287

**Published:** 2023-02-14

**Authors:** Qian Wang, Yue Jiang, Li Lin, Sheng Li, Jiagao Lv, Jun Chen

**Affiliations:** ^1^ Department of Internal Medicine, Division of Cardiology, Tongji Hospital, Tongji Medical College, Huazhong University of Science and Technology, Wuhan, China; ^2^ Division of Cardiothoracic and Vascular Surgery, Tongji Hospital, Tongji Medical College, Huazhong University of Science and Technology, Wuhan, China

**Keywords:** cardiac, atrial tumor, clinical characteristics, myxoma, primary tumor

## Abstract

**Background:**

Primary atrial tumors are relatively rare and predominantly benign. However, some atrial tumors may be malignant and are associated with poor outcome. Currently, it is hard to determine the malignance of atrial tumors by preoperative clinical presentation or by echocardiography. We aimed to report the difference in the clinical characteristics of patients with benign and malignant atrial tumor.

**Methods:**

This was a single-center retrospective study. A total of 194 patients with primary atrial tumor admitted to our center between 2012 and 2021 were included. The clinical characteristics of patients with benign and malignant tumor were compared.

**Results:**

Benign and malignant tumor accounted for 93% (*n* = 180) and 7% (*n* = 14) of the total patients, respectively. Malignant atrial tumor tended to occur in younger patients (*P* < 0.05), was more likely to be located at the right atrium (*P* < 0.05), and tended to attach to the atrial wall or valve instead of the atrial septum. Fever symptoms were more common in patients with malignant tumors than in patients with benign tumors (*P* < 0.05). Compared to benign tumor, patients with malignant atrial tumor also demonstrated higher rates of fever, lower rates of increasing fibrinogen, increased blood glucose (*P* < 0.05), significantly longer prothrombin time, and lower prothrombin activity (*P* < 0.05). Patients with malignant primary atrial tumor had higher mortality rate, tumor metastasis rate, and tumor recurrence rate than patients with benign primary atrial tumor (*P* < 0.05).

**Conclusion:**

We compared the clinical characteristics of patients with benign and malignant atrial tumor. These findings provide valuable information to preoperatively determine the malignance of atrial tumor and thus guide surgical treatment.

## Introduction

Primary cardiac tumors are rare with an estimated incidence of 1,380/100 million individuals in the general population (1). Benign tumors account for the majority of them and have a favorable surgical prognosis, while malignant cardiac tumors are usually aggressive and are associated with poor outcome (2, 3). The atrial is a common site where cardiac tumor occurs. Even benign atrial tumor, due to its size and location, may lead to severe hemodynamic or arrhythmic consequence. The determination of the malignance is essential for surgical treatment of atrial tumors. Histopathological examination is the gold standard to diagnose primary atrial tumor (4), but it is not a practical way for preoperative diagnosis of malignant atrial tumors due to the difficulty in obtaining a biopsy of cardiac tumors before surgery. Thus, the manifestations and echocardiography findings of patients are of more practical value for the diagnosis and treatment of atrial tumors. The symptoms of patients with atrial tumor have been reported in previous studies, ranging from asymptomatic detection to cardiac obstructive symptoms, dyspnea, and constitutional symptoms (5, 6). However, there is still a lack of data to comprehensively compare the clinical characteristics associated with benign or malignant atrial tumors.

In this study, we included patients with benign or malignant tumors and comprehensively compared their demographic features, laboratory test results, and echocardiography findings. We also compared their surgical information and in-hospital and long-term outcome, aiming to provide a better understanding about the clinical characteristics, which may help to improve the clinical diagnosis and treatment of atrial tumor.

## Methods

### Patients

In this retrospective study, we included 194 patients who received cardiac surgery due to atrial tumor at our hospital from 2012 to 2021. Primary cardiac tumor was diagnosed by histopathological examination on specimens obtained during surgery. Patients' clinical, radiologic, and pathologic characteristics, including age, gender, initial symptoms, comorbidities, echocardiographic results, electrocardiogram results, and pathological diagnosis, were collected retrospectively from electronic medical records through standardized data collection forms. The classification of benign and malignant cardiac tumors in these patients was based on the fourth edition of the WHO Classification of Tumors. Primary cardiac tumors refer to those occurring at the cardiac tissue, and do not include cardiac metastases from tumors in other organs (2). Patients with secondary cardiac tumors or tumors not located in the atrial region were excluded. Patients who met the inclusion criteria were consecutively recruited. Follow-up information was obtained through outpatient and inpatient medical records or telephone calls.

### Statistical analysis

Statistical analyses were performed in the SPSS ® software (version 28, IBM). Categorical variables were described as frequency and percentage (%), and the chi-square (*χ*^2^) test or the Fisher exact test was used to determine the difference between two groups. For continuous variables, the normality test was first performed. If continuous variables demonstrated normal distribution, the mean (Standard Deviation, SD) was used for description, and the *t* test was used to compare the difference; if the normal distribution is not met, the median (Interquartile range, IQR) was used for statistical description, and Mann–Whitney test was used for statistical test. All statistical tests were bilateral tests, and results with *P* < 0.05 were considered to be significant.

## Results

### General data

**Figure 1:**
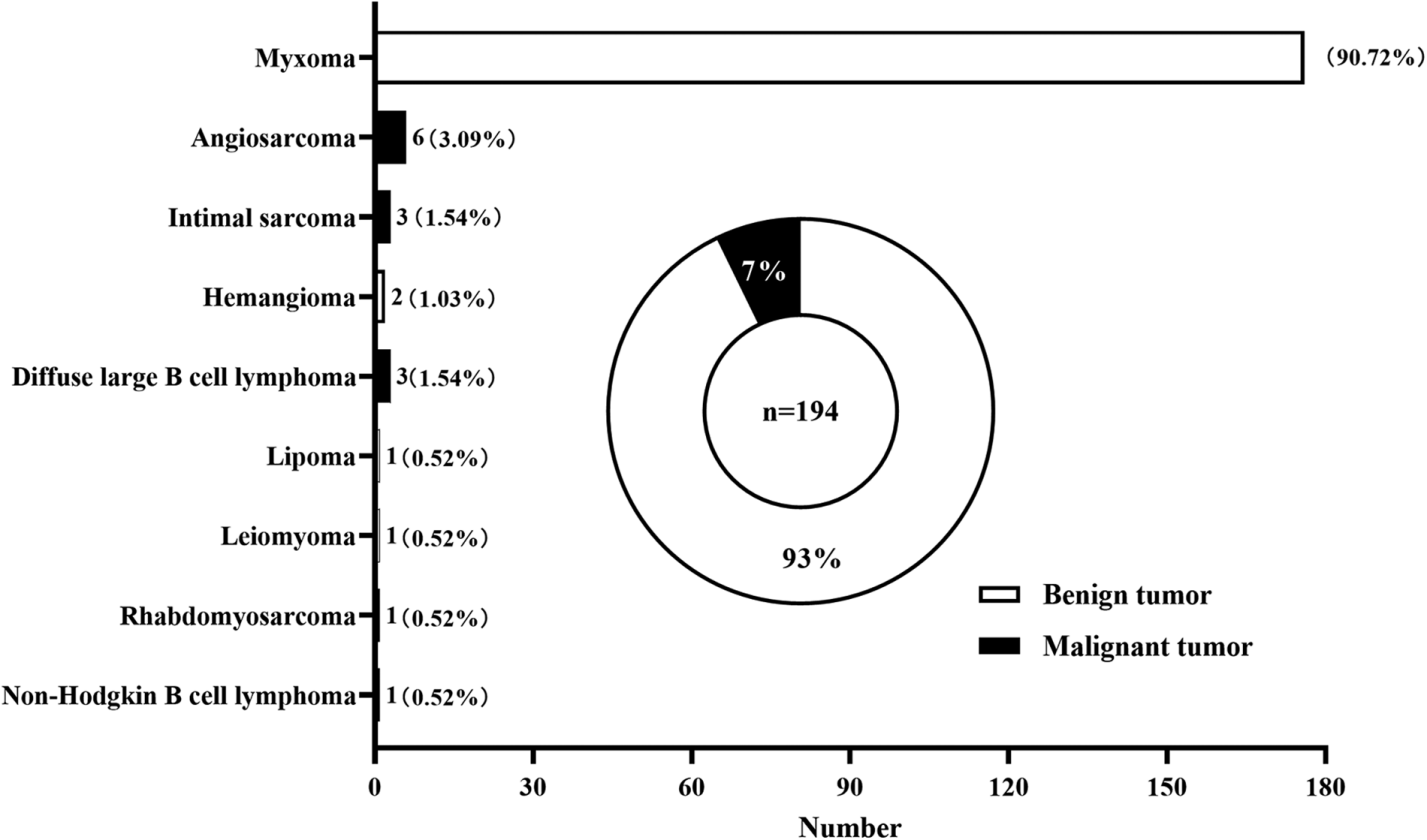
distribution of primary cardiac tumors located in the atrium diagnosed and treated at division of cardiothoracic and vascular surgery of tongji hospital between 2012 and 2021.

A total of 194 patients with primary atrial tumor were included in this single-center study, including 180 (93%) patients with benign tumor and 14 (7%) patients with malignant tumor (Figure 1). Myxoma was the most common type of benign tumors, accounting for 90.72% of the total tumors. Other kinds of benign tumors included hemangioma (1.03%), lipoma (0.52%), and leiomyoma (0.52%). Among malignant atrial tumors, angiosarcoma (3.09%) had the highest incidence rate, followed by intimal sarcoma (1.54%) and diffuse large B cell lymphoma (1.54%). Rhabdomyosarcoma (0.52%), non-Hodgkin B cell lymphoma (0.52%) were also seen in atrial tumors.

The average age of patients with primary malignant atrial tumor were 40 years, which was significantly younger than that of patients with benign atrial tumor (52 years; *P* < 0.05) (Table 1). Female patients accounted for 66.7% in the benign tumor group and 50% malignant tumor tumor group. No significant difference was noted in gender distribution between the two groups (*P* > 0.05). In terms of tumor site, benign atrial tumors were more likely to occur in the left atrium (89.4%; *P* < 0.001), while malignant tumors tended to present in the right atrium (57.1%; *P* < 0.001). Bilateral atrial tumors were only observed in three patients in the benign group. Blood pressure and heart rate measurements showed no significant difference between the benign and malignant groups.

**Table 1:** comparison of clinical characteristics between patients with primary atrial benign tumors and primary atrial malignant tumors.

Clinical characteristics	Benign tumor (*n* = 180)	Malignant tumor (*n* = 14)	*P* value
Age, mean (SD), years	52 (12)	40 (11)	<0.001
Sex			0.247*
Female (%)	120 (66.7)	7 (50.0)	
Male (%)	60 (33.3)	7 (50.0)	
**Tumor sites**			
Left atrium (%)	161 (89.4)	6 (42.9)	<0.001*
Right atrium (%)	16 (8.9)	8 (57.1)	<0.001*
Biatrium (%)	3 (1.7)	0 (0)	1**
SBP, median (IQR), mmHg	120 (15)	110 (29)	0.088^#^
DBP, median (IQR), mmHg	75 (11)	74 (17)	0.283^#^
HR, median (IQR), bpm	80 (11.5)	87 (22)	0.088^#^
BMI	21.8 (4.6)	19.9 (5.05)	0.125^#^

*P* values comparing benign tumors group and malignant tumor group were from *t* test.

* *P* values were calculated by *χ*^2^ test.

** *P* values were calculated by Fisher exact test.

^#^ *P* values were from Mann–Whitney test. SBP, Systolic Blood Pressure; DBP, Diastolic Blood Pressure; HR, Heart Rate; bpm,  beats per minute; BMI, Body Mass Index; SD, Standard Deviation; IQR, Interquartile Range.

### Clinical symptoms

**Figure 2:**
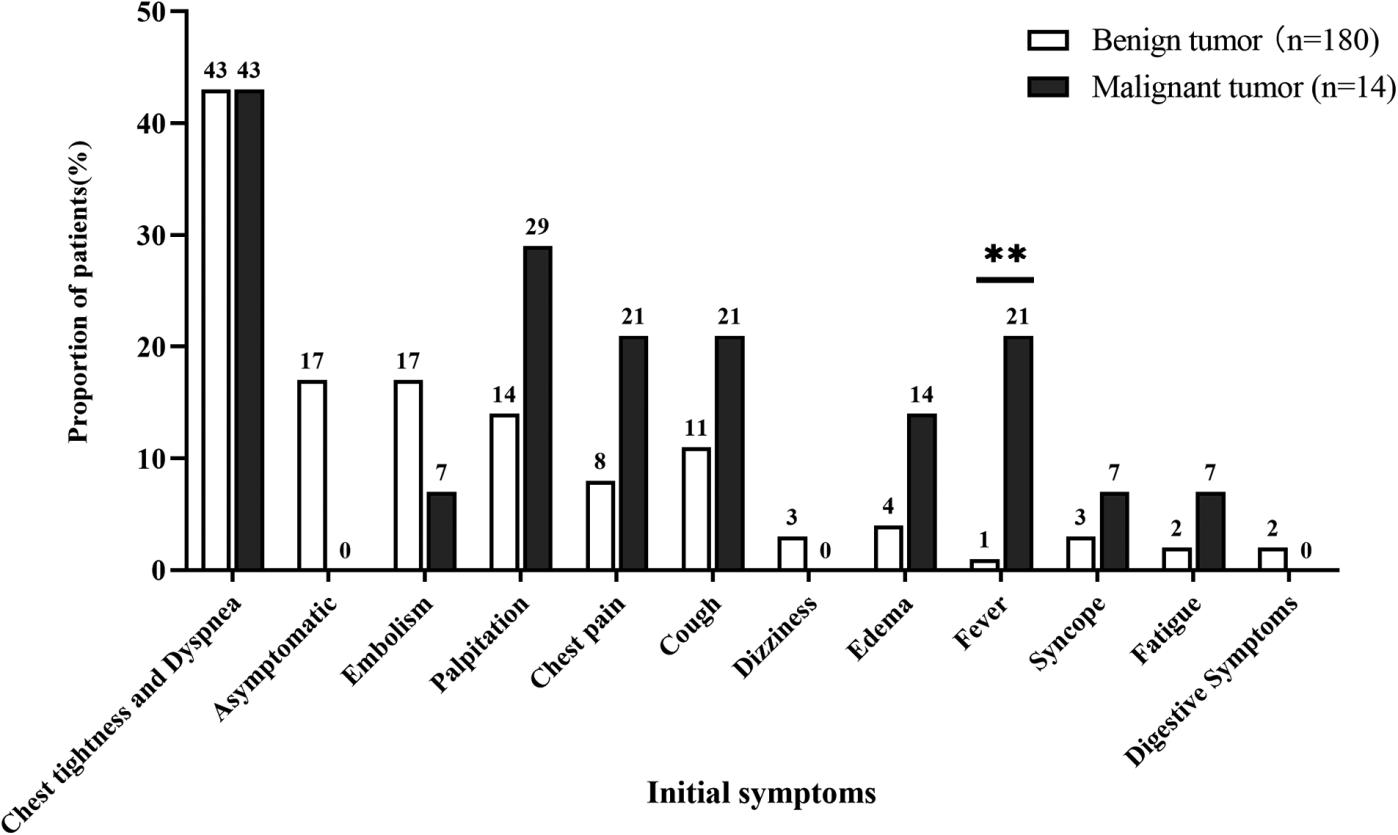
Comparison of initial symptoms between patients with benign and malignant primary atrial tumors. ** represets *P* < 0.01.

Patients with atrial tumor presented various initial symptoms, which mainly depended on tumor size and site, regardless of tumor type. Chest tightness and dyspnea were the most common symptoms of patients with atrial tumor (Figure 2). Thirty-one (17%) patients with benign tumor were asymptomatic, and masses in the atrium were found through imaging tests during physical examination. Embolism was a life-threatening complication of cardiac tumor (7, 8), and 30 (17%) benign atrial tumors and 1 (7%) malignant atrial tumor were first diagnosed after the onset of cerebral or peripheral vasculature embolism. Fever accounted for a rate of 21% in the malignant tumor group, which was greatly higher than that in the benign tumor group (1%; *P* < 0.05). Palpitation, chest pain, cough, dizziness, edema, syncope, fatigue, and digestive symptoms were also observed in patients with primary atrial neoplasm.

### Blood routine characteristics

Blood assay results were available in 193 of all the patients before surgery, and coagulation tests were performed in 187 patients. The rate of patients with increased blood glucose level was significantly higher in the malignant tumor group (78.5%) than that in the benign tumor group (43.3%; *P* = 0.001) (Table 2). Remarkably, significantly shorter prothrombin time (13.8 s VS 14.7 s; *P* < 0.05) and lower prothrombin activity (78% vs. 90%; *P* < 0.05) were observed in patients with malignant atrial tumor. The increase in fibrinogen level was more common in patients with benign atrial tumor than in patients with malignant tumor (*P* < 0.05).

**Table 2:** The blood routine characteristics of patients with primary atrial tumor.

Variables	Benign tumor (*n* = 180)	Malignant tumor (*n* = 14)	*P* value
**Leucocytes ( ×10^9^ per L; normal range 3.5–9.5)**			
Median (IQR)	6.3 (2.3)	7.2 (1.9)	0.110^#^
Increased (%)	15 (8.3)	1 (7.1)	1**
Decreased (%)	7 (3.9)	0	1**
**Red blood cell ( × 10^12^per L; normal range 4.3–5.8)**			
Median (IQR)	4.3 (7)	4.1 (1.1)	0.711^#^
Increased (%)	1 (0.6)	1 (7.1)	0.139**
Decreased (%)	98 (54.4)	10	0.271**
**Haemoglobin [g/l; normal range: male (130.0–175.0), female (115.0–150.0)]**			
Median (IQR)	121 (25.5)	118 (26.0)	0.789^#^
Increased (%)	0	0	
Decreased (%)	81 (45.0)	8 (57.1)	0.380*
**Platelets ( ×10^9^ per L; normal range 125.0–350.0)**			
Median (IQR)	231 (116.5)	220 (132.0)	0.430^#^
Increased (%)	15 (8.3)	3 (21.4)	0.127**
Decreased (%)	13 (7.2)	0	0.604**
**Blood glucose (mmol/l; normal range 4.11–6.05)**			
Median (IQR)	5.26 (1.10)	6.19 (2.92)	0.111^#^
Increased (%)	78 (43.3)	11 (78.5)	0.013**
Decreased (%)	1 (0.6)	0	1**
**Prothrombin Time (s; normal range 11.5–14.5)**			
Median (IQR)	13.8 (1.35)	14.7 (1.6)	0.024^#^
Increased (%)	50 (27.8)	6 (42.8)	0.230*
Decreased (%)	0	0	
**Prothrombin activity (%; normal range 75–125)**			
Median (IQR)	90 (17)	78 (20)	0.026^#^
Increased (%)	0	0	
Decreased (%)	13 (7.2)	2 (14.3)	0.296**
**Fibrinogen (g/l; normal range 2–4)**			
Median (IQR)	4.1 (1.9)	3.2 (1.8)	0.103^#^
Increased (%)	95 (52.8)	1 (7.1)	0.001**
Decreased (%)	1 (0.6)	0	1**
**Activated Partial Thrombin Time (s; normal range 29–42)**			
Median (IQR)	38.5 (7.3)	35.9 (9.2)	0.609^#^
Increased (%)	51	2	0.358**
Decreased (%)	4	0	1**

* *P* values were calculated by *χ*^2^ test.

** *P* values were calculated by Fisher exact test.

^#^ *P* values were from Mann–Whitney test. IQR, Interquartile Range.

### Echocardiographic and electrocardiogram characteristics

**Figure 3:**
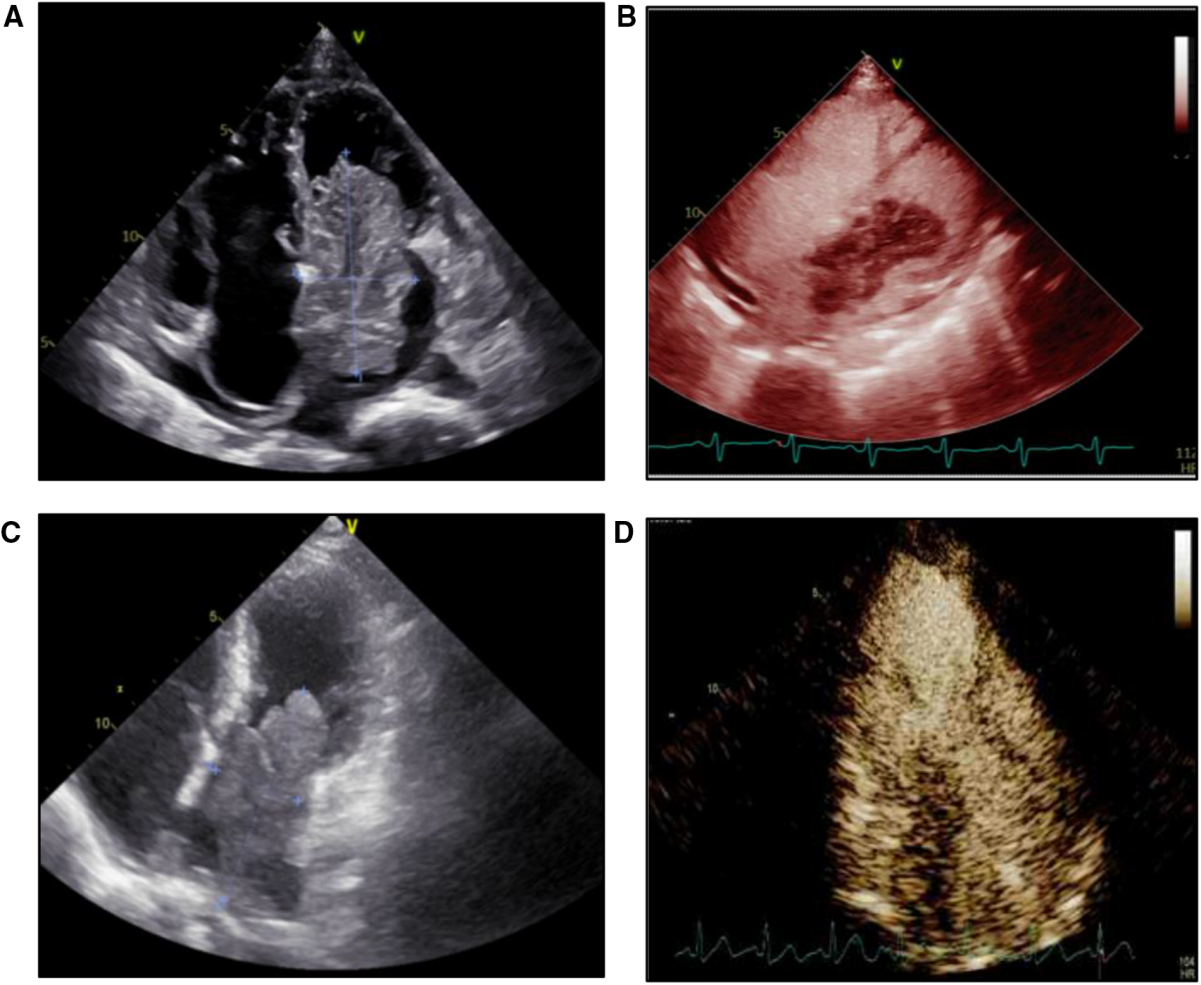
Echocardiography and microbubble contrast-enhanced echocardiography images of patients with benign or malignant primary atrial tumor. (**A**) Echocardiography image of a patient with cardiac myxoma. (**B**) Microbubble contrast-enhanced echocardiography image of a patient with cardiac myxoma. (**C**) Echocardiography image of a patient with intimal sarcoma. (**D**) Microbubble contrast-enhanced echocardiography image of a patient with intimal sarcoma.

Tumor size and activity were similar between the two groups (Table 3). For the tumor attaching site, over 90% of benign tumors were attached to the atrial septum, compared to only 14% in the malignant tumor group (*P* < 0.05). Representative echocardiographic images of benign and malignant primary atrial tumors were shown in Figure 3A–C. Echocardiography with ultrasound-enhancing agents is an important tool for the evaluation of the relative perfusion of a cardiac tumor. Malignant atrial tumors often present abundant blood supply and demonstrate greater enhancement than the adjacent myocardium, while benign atrial tumors usually have poor blood supply (Figure 3B–D). Cardiac computed tomography (CT) and cardiovascular magnetic resonance (CMR) are also important for the assessment of cardiac tumors (Supplementary Figure S1). No difference was observed in the rate of electrocardiogram change between the two groups, including non-specific T wave change, ST segment changes, atrial arrhythmias or fibrillations, tachycardia and bradycardia. Pericardial effusion was present in 5 patients (35.7%) of the malignant group, which was higher than that in the benign group (13.9%) (*P* < 0.05). These differences in symptoms, locations, complications, laboratory and imaging manifestations between benign and malignant atrial tumor patients may provide valuable information for the preoperative diagnosis.

**Table 3:** Echocardiographic and electrocardiogram characteristics of primary atrial tumor patients.

Variables	Benign tumor (*n* = 180)	Malignant tumor (*n* = 14)	*P* value
**Echocardiographic variables**			
Tumor long diameter, median (IQR), cm	4.9 (3.0)	5.8 (3.1)	0.159^#^
Tumor Transverse Diameter, median (IQR), cm	3.2 (1.5)	2.9 (2.1)	0.922^#^
Tumor activity			0.225**
Activity is confined to the atrium (%)	108 (60)	11 (78)	
Travels between atrium and ventricle (%)	72 (40)	3 (22)	
Tumor attachment site			<0.001**
Atrial septum (%)	164 (91)	2 (14)	
Atrial wall or valve (%)	16 (9)	12 (86)	
Left atrium diameter, median (IQR), mm	37 (11)	34 (8)	0.257^#^
LVEF, median (IQR), %,	66 (6)	65 (11)	0.509^#^
**Electrocardiogram variables**			
T wave change (%)	25 (13.9)	1 (7.1)	0.698**
ST segment changes (%)	11 (6.1)	3 (21.4)	0.068**
Atria arrhythmias or fibrillations (%)	17 (9.4)	1 (7.1)	1**
Premature ventricular contraction (%)	3 (1.7)	0 (0)	1**
Tachycardia (%)	16 (8.9)	3 (21.4)	0.145**
Bradycardia (%)	4 (2.2)	2 (14.3)	0.062**
**Complications**			
Pulmonary hypertension (%)	37 (20.5)	0 (0)	0.076**
Pericardial effusion (%)	25 (13.9)	5 (35.7)	0.048*
Valvular disease (%)	29 (16.1)	1 (7.1)	0.700**

* *P* values were calculated by *χ*^2^ test.

** *P* values were calculated by Fisher exact test.

^#^ *P* values were from Mann–Whitney test. LVEF,  Left Ventricular Ejection Fraction; IQR, Interquartile Range.

### Operation information

**Figure 4:**
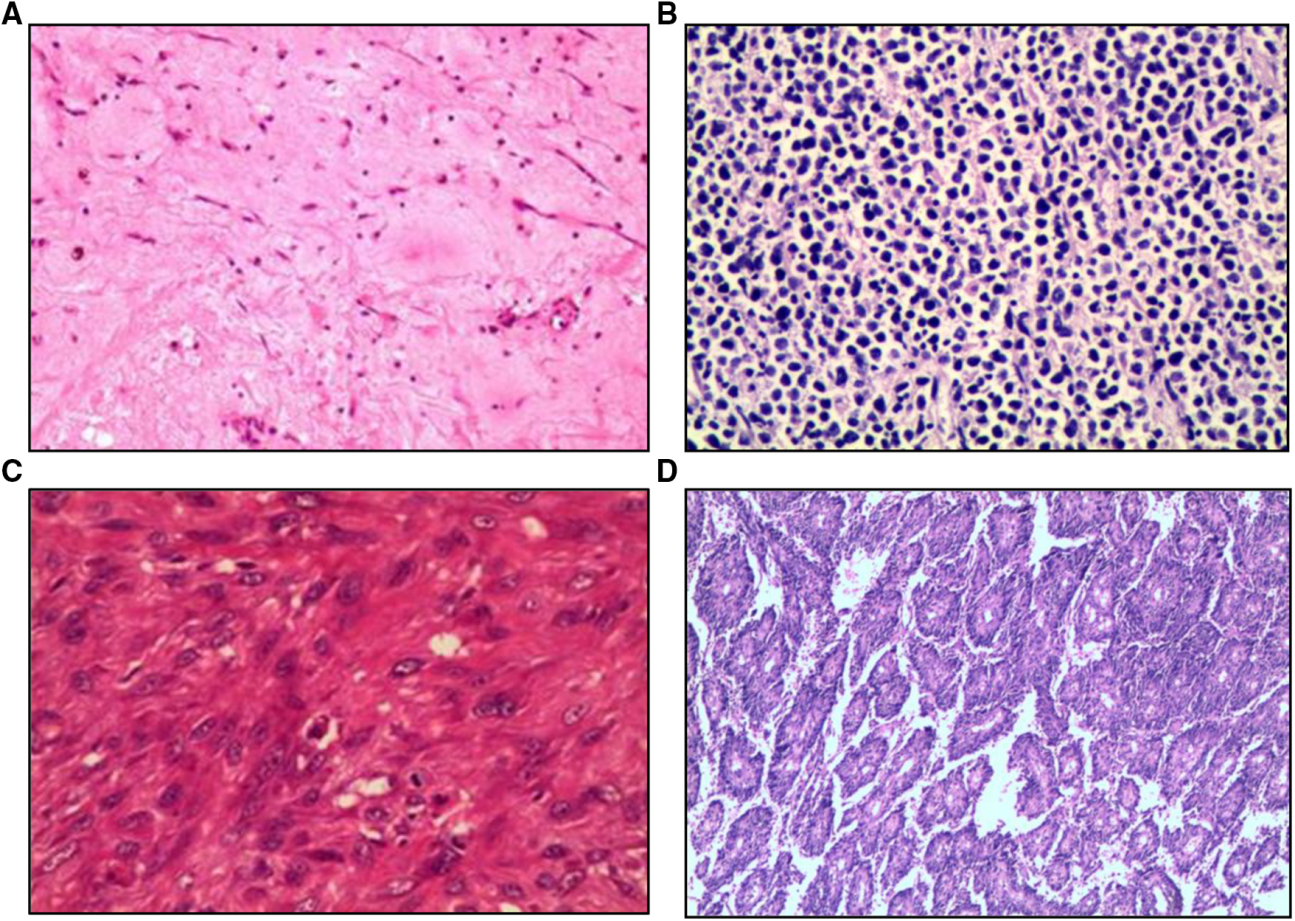
Pathological section images of patients with benign or malignant primary atrial tumor. (**A**) Cardiac myxoma. (**B**) Diffuse large B cell lymphoma. (**C**) Intimal sarcoma. (**D**) Angiosarcoma.

All procedures were performed through a standard median sternotomy. Cannulation strategy was similar between the two groups (Table 4). All benign atrial tumors were completely resected. Nine patients (64.3) with malignant atrial tumor underwent complete resection including one with allogeneic orthotopic heart transplantation and another with autogenous heart graft. Five patients with malignant atrial tumor underwent partial resection. Seventy-eight patients (43.3%) with benign primary atrial tumor required heart reconstruction after tumor removal with prosthetic material (Autologous pericardial patch, *n* = 57 (31.7). Bovine pericardial patch, *n* = 7 (3.9). Dacron patch, *n* = 14 (7.8)). Two patients (14.3%) with malignant primary atrial tumor underwent patch reconstruction and both with autologous pericardial patch. Fourteen patients with benign primary atrial tumor underwent concomitant cardiac surgical procedure including coronary artery bypass grafting (1.1%), mitral valve repair (2.8%), and tricuspid valve repair (3.9%). One patient with intimal sarcoma underwent coronary artery bypass grafting in addition to tumor resection. The pathological sections were used to judge the classification of primary atrial tumor (Figure 4). During the operation, cardiopulmonary bypass time and operation time of the malignant tumor group were significantly longer than that of the benign tumor group (63.5 (57.0) min vs. 52 (20.5) min,4.04 (0.7) hour vs. 3.92 (1.1) hour, *P* < 0.05) (Table 4).

**Table 4:** Operation characteristics of primary atrial tumor patients.

Variables	Benign tumor (*n* = 180)	Malignant tumor (*n* = 14)	*P* value
**Cannulation strategy**			
Bicaval (%)	160 (88.9)	11 (78.6)	0.221**
Two-stage (%)	14 (7.8)	0 (0)	0.604**
Femoral Vessel (%)	6 (3.3)	1 (7.1)	0.413**
Complete resection	180 (100)	9 (64.3)	<0.001**
Allogeneic orthotopic heart transplantation (%)	0 (0)	1 (7.1)	<0.001**
Autogenous heart graft (%)	0 (0)	1 (7.1)	<0.001**
Partial resection	0 (0)	5 (35.7)	<0.001**
**Technique**			
Simple tumor resection (%)	102 (56.7)	12 (85.7)	0.065*
Patch reconstruction (%)	78 (43.3)	2 (14.3)	0.065*
**Patch material**			
Autologous pericardial patch (%)	57 (31.7)	2 (14.3)	0.234**
Bovine pericardial patch (%)	7 (3.9)	0 (0)	1.000**
Dacron patch (%)	14 (7.8)	0 (0)	0.604**
**Concomitant procedures**			
CABG (%)	2 (1.1)	1 (7.1)	0.202**
Mitral valve repair (%)	5 (2.8)	0 (0)	1.000**
Tricuspid valve repair (%)	7 (3.9)	0 (0)	1.000**
**Duration**			
CPB time, median (IQR), min	52 (20.5)	63.5 (57.0)	0.008^#^
Operation time, median (IQR), hour	3.92 (1.1)	4.04 (0.7)	0.030^#^

* *P* values were calculated by *χ*^2^ test.

** *P* values were calculated by Fisher exact test.

^#^ *P* values were from Mann–Whitney test. CABG, coronary artery bypass grafting; CPB, cardiopulmonary bypass; IQR, Interquartile Range.

### Postoperative outcomes

Postoperative outcomes are shown in Table 5. Left ventricular ejection fraction after surgery and postoperative hospital stay time were similar in both groups. All patients survived to hospital discharge. Follow-up was obtained in 67.2% of benign tumor patients and in 64.2% of malignant tumor patients. Patients with malignant primary atrial tumor had higher mortality rate, tumor metastasis rate, and tumor recurrence rate than patients with benign primary atrial tumor (22.2% vs. 2.4%, 22.2% vs. 0%, 22.2% vs. 0.8%). Follow-up information for all the patients with malignant primary atrial tumor are shown in Supplementary Table S1. Two patients with angiosarcoma received postoperative chemotherapy. One patient with intimal sarcoma underwent postoperative chemotherapy combined with radiotherapy, and two patients received postoperative chemotherapy. One patient with rhabdomyosarcoma and one patient with angiosarcoma died within one year after surgery.

**Table 5:** Postoperative results of primary atrial tumor patients.

Variables	Benign tumor (*n* = 180)	Malignant tumor (*n* = 14)	*P* value
**Early outcomes**			
LVEF after surgery	61 (7)	61 (6)	0.613^#^
Postoperative hospital stay time, median (IQR), day	14 (4)	16 (7)	0.584^#^
In-hospital death (%)	0 (0)	0 (0)	
**Postoperative complications**			
Atrial fibrillation (%)	2 (1.1)	0 (0)	1.000**
Pneumonia (%)	5 (2.7)	1 (7.1)	0.366**
Pleural effusion (%)	6 (3.3)	0 (0)	1.000**
Pericardial effusion (%)	4 (2.2)	0 (0)	1.000**
Stroke (%)	1 (0.6)	0 (0)	1.000**
Fat liquefaction of incision (%)	1 (0.6)	0 (0)	1.000**
**Late outcomes**			
Number of patients followed-up	121	9	
Follow-up time, median (IQR), month	54.8 (48.1)	15.0 (15.2)	
Death after discharge (%)	3 (2.4)	2 (22.2)	0.038**
Cardiac tumor metastasis (%)	0 (0)	2 (22.2)	0.004**
Cardiac tumor recurrence (%)	1 (0.8)	2 (22.2)	0.012**

** *P* values were calculated by Fisher exact test.

^#^ *P* values were from Mann–Whitney test. LVEF,  Left Ventricular Ejection Fraction; IQR, Interquartile Range.

## Discussions

This single-center retrospective study included 194 patients with primary atrial tumor between 2012 and 2021. Patients were divided into two groups according to whether the tumor was benign or malignant. We found that the average age of patients with benign tumors was older than those with malignant tumors. Benign atrial tumors mostly occurred in middle-aged and elderly patients (7, 8), while malignant atrial tumors tend to occur in younger people, which may be due to the slower progression of benign tumors and the aggressiveness of malignant tumors. Tumors located at the right atrium were more likely to be malignant than those located at the left atrium. However, malignant tumors may also arise from the left atrium and need to be further differentiated from benign tumors.

Previous studies have shown that the symptoms of patients with cardiac tumor are nonspecific, depending on tumor size and site, regardless of tumor type (5, 9–11). However, we found that patients with malignant atrial tumors had a higher proportion of fever than patients with benign atrial tumors. Neoplastic fever is a noninfectious fever directly related to tumors. It can be diagnosed when infection is ruled out and antibiotic treatment is ineffective. Malignant tumors commonly leading to fever include soft-tissue sarcoma, Hodgkin, and non-Hodgkin lymphomas (12). These malignancies are the main components of primary malignant atrial tumors. Fever may be a valuable symptom to inform the malignancy of atrial tumors.

In terms of blood biochemistry, we found patients with malignant atrial tumor tended to have elevated blood glucose level, while increased fibrinogen level were more common in patients with benign atrial tumor. The mechanisms underlying elevated blood glucose in malignant atrial tumor and increased blood fibrinogen level in benign atrial tumor are currently unknown. The prothrombin time was shorter and the prothrombin activity was higher in patients with benign atrial tumor. Thus, the risk of thrombosis in patients with benign atrial tumor could be greater. As embolism is a life-threatening complication of cardiac tumor, great attention should be paid to the blood coagulation state in patients with benign atrial tumor.

Before the popularization of echocardiography in clinical practice, primary cardiac tumors are rarely found, and most of them are reported in autopsy series. With the development of medical imaging technology and the enhancement of people's health awareness, increasingly atrial tumors have been discovered and finally diagnosed (13). Echocardiography is a primary tool for the assessment of atrial tumor, providing an accurate assessment of tumor size, location, and attachment site and should assist in surgical planning (14, 15). Compared with benign atrial tumors, the specific characteristics of malignant atrial tumors on echocardiography are still unclear. We found that most benign atrial tumors were located at the atrial septum, while most malignant atrial tumors were attached to the atrial wall or valve. Pericardial effusion was more common in patients with malignant tumor. Attachment to the atrial wall or valve and pericardial effusion could be two informative characteristics for diagnosing malignant atrial tumor. Echocardiography with ultrasound-enhancing agents is becoming an increasingly important tool to evaluate the relative perfusion of a cardiac tumor (7). Malignant tumors tend to have significantly more feeding arteries than benign tumors and present greater enhancement than the adjacent myocardium in echocardiography with ultrasound-enhancing agents (7). Cardiac CT is useful in the evaluation of calcified, global chest assessment, and tumor staging, which could be helpful in surgical planning (16, 17). CMR could assess cardiac tumor characteristics including dimensions, location, morphology, extension, homogeneity, presence of infiltration in the surrounding tissues, and s pathophysiological properties. However, CMR have lower temporal resolution and longer acquisition time than echocardiography, which could account for the limited availability of CMR (18, 19). When malignant atrial tumor is suspected in patients, further diagnostic tests are required. 18F-fluorodeoxyglucose positron emission tomography/computed tomography (FDG-PET/CT) test may provide higher diagnostic value, as it possesses a sensitivity of over 90% in distinguishing benign and malignant cardiac tumors. Besides, the metastatic state of the malignant tumor can also be evaluated by PET-CT (20). Such method combined the clinical manifestations can provide a comprehensive prognostic information for patients with malignant atrial tumor. Patients with unresectable primary atrial tumors and no evidence of metastasis, surgical treatment is generally tended to improve the prognosis. The surgical approach was based on characteristics of the tumor. Some approaches including heart transplantation or autogenous heart graft should also be considered. The classification of primary atrial tumor as benign or malignant is important for the prediction of prognosis. Our results found that surgical resection of primary benign atrial tumor has good long-term results, while the prognosis of malignant primary atrial tumor is poor. Benign tumors are asymptomatic when the volume is small, and the growth rate of the tumor in the heart is generally slow. Malignant atrial tumors are highly aggressive and progress quickly (21). If the patient with malignant cardiac tumor cannot receive effective treatment, the outcome will be very poor, with overall survival of only several months (22). The treatment of malignant atrial tumors remains a challenge for clinicians, and a comprehensive treatment strategy should be made by interdisciplinary teams including oncologist, cardiologists and cardiac surgeons (23).

### Limitations

One major limitation of our study is that it was a single-center study, and the sample size of patients with malignant atrial tumor are small. More patients could be included from multi-centers to evaluate the risk factors and outcomes of atrial tumors. In addition, we only analyzed the clinical characters between benign and malignant atrial tumors, and the molecular mechanism by which malignant atrial tumor develops need further basic research. Last but not least, many patients were lost to follow-up, which could affect the assessment of the prognosis of patients with primary atrial tumor.

## Conclusions

This single center retrospective study revealed that patients with malignant atrial tumors are commonly accompanied by fever. These patients tend to be younger, have longer prothrombin time, lower prothrombin activity, and increased blood glucose level than patients with benign atrial tumor. On echocardiogram, malignant atrial tumors generally attach to the atrial septum or valve and have pericardial effusion. Patients with malignant primary atrial tumors had less favorable prognosis. The differences in preoperative clinical features between patients with benign and malignant atrial tumors may provide valuable reference for presumptive diagnosis.

## Data Availability

The original contributions presented in the study are included in the article/**Supplementary Material**, further inquiries can be directed to the corresponding author.
