# Supplementary material for: Clinical characteristics of primary atrial tumor and their diagnostic value: A retrospective study of 10 years
Source: Front Surg. 2023 Feb 14;10:1097287. doi: 10.3389/fsurg.2023.1097287 (PMC9971564; doi:10.3389/fsurg.2023.1097287)
Supplement: Supplementary file 1 [file Datasheet1.pdf]

Supplementary Material

1. Supplementary Figure

Supplementary Figure 1

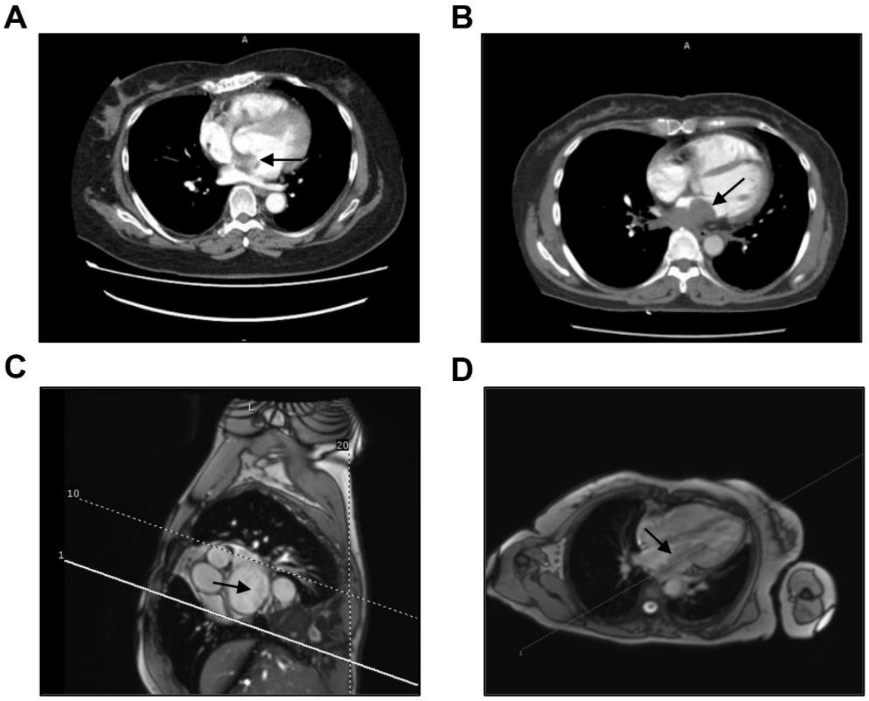

Supplementary Figure 1. Cardiac computed tomography (CT) and cardiovascular magnetic resonance (CMR) images of patients with benign or malignant primary atrial tumor. (A) CT image of a patient with cardiac myxoma. (B) CT image of a patient with intimal sarcoma. (C) CMR image of a patient with intimal sarcoma. (D) CMR image of a patient with intimal sarcoma.

2. Supplementary Table

Supplementary Table 1. Follow-up information for all the patients with malignant primary atrial tumor.

Tumor type	Operation	Other therapy	Prognosis
Angiosarcoma	Partial resection	Chemoteraphy (ifosfamide +Adriamycin)	Lost to follow-up
	Partial resection	/	Lost to follow-up
	Partial resection	None	Death within one year after surgery
	Complete resection	Chemoteraphy (nab-paclitaxel)	Metastasis and recurrence within one year after surgery
	Complete resection	/	Lost to follow-up
	Allogeneic orthotopic heart transplantation	None	Metastasis within one year after surgery
Intimal sarcoma	Complete resection	Chemoteraphy (irinotecan+etoposide)	No recurrence or metastasis，follow-up time 32 months
	Complete resection	Chemoteraphy (ifosfamide+adriamycin) and radiotherapy	No recurrence or metastasis，follow-up time 18 months
	Autogenous heart graft	Chemoteraphy (gemcitabine-docetaxel)	Recurrence within two years after surgery
Diffuse large B cell lymphoma	Complete resection	None	No recurrence or metastasis，follow-up time 17 months
	Complete resection	None	No recurrence or metastasis，follow-up time 20 months
	Complete resection	/	Lost to follow-up
Rhabdomyosarcoma	Partial resection	None	Death within one year after surgery
Non-Hodgkin B cell lymphoma	Partial resection	/	Lost to follow-up
